# Chagas Disease in Pregnant Women from Endemic Regions Attending the Hospital General de Mexico, Mexico City

**DOI:** 10.3390/tropicalmed7010008

**Published:** 2022-01-11

**Authors:** Indira Chakravarti, Monica Miranda-Schaeubinger, Adriana Ruiz-Remigio, Carlos Briones-Garduño, Edith A. Fernández-Figueroa, Concepción Celeste Villanueva-Cabello, Alejandra Borge-Villareal, Yadira Bejar-Ramírez, Alejandro Pérez-González, César Rivera-Benitez, Eyal Oren, Heidi E. Brown, Ingeborg Becker, Robert H. Gilman

**Affiliations:** 1 Department of International Health, Johns Hopkins School of Public Health, Baltimore, MD 21215, USA; rgilman1@jhu.edu; 2 Centro de Medicina Tropical, División de Investigación, Facultad de Medicina, Universidad Nacional Autónoma de México, Ciudad de México 04510, Mexico; ruizad11@hotmail.com (A.R.-R.); eafernandez@inmegen.edu.mx (E.A.F.-F.); concepcioncelestevillanueva@gmail.com (C.C.V.-C.); 3 Servicio de Ginecología y Obstetricia, Hospital General de México “Dr. Eduardo Liceaga”, Ciudad de México 06720, Mexico; drcarlosbriones@yahoo.com.mx (C.B.-G.); aborge.villarreal20@gmail.com (A.B.-V.); 4 Computational and Integrative Genomics, National Institute of Genomic Medicine, Ciudad de México 14610, Mexico; 5 Banco de Sangre, Hospital General de México “Dr. Eduardo Liceaga”, Ciudad de México 06720, Mexico; yadira_bejarr@yahoo.com.mx (Y.B.-R.); jalex_perezg@hotmail.com (A.P.-G.); 6 Servicio de Infectología, Hospital General de México “Dr. Eduardo Liceaga”, Ciudad de México 06720, Mexico; rivera.cesar85@gmail.com; 7 Division of Epidemiology and Biostatistics, School of Public Health, San Diego State University, San Diego, CA 92182, USA; eoren@sdsu.edu; 8 Mel and Enid Zuckerman College of Public Health, University of Arizona, Tucson, AZ 85724, USA; heidibrown@arizona.edu

**Keywords:** Chagas disease, congenital Chagas disease, *Trypanosoma cruzi*, Chagas disease diagnosis

## Abstract

*Trypanosoma cruzi* infection leads to Chagas disease (CD), a neglected tropical infection of significant public health importance in South and Central America and other, non-endemic, countries. Pregnant women and their children are of particular importance to screen as *T. cruzi* can be transmitted vertically. The objective of this study was to screen for *T. cruzi* infection among pregnant women from endemic areas seen at the Hospital General de Mexico for prenatal care, so that they and their children may be quickly connected to CD treatment. Pregnant women were recruited through the hospital prenatal clinic and screened for *T. cruzi* infection using a series of serological and molecular tests. Of 150 screened patients, mean age 26.8 (SD 6.4), 30 (20.0%) were positive by at least one diagnostic test. Of these, only nine (6%) were positive as determined by PCR. Diagnosis of chronic CD is difficult in endemic places like Mexico due to the limitations of current commercially available diagnostic tests. Further evaluation of diagnostic performance of various assays could improve current CD diagnostic algorithms and proper care management in these regions. Genetic variability in the parasite may also play a role in the differing assay performances seen in this study, and this may be a valuable avenue of further research.

## 1. Introduction

Chagas disease (CD) is a neglected tropical disease caused by the parasite *Trypanosoma cruzi*. Over six million people worldwide are affected, mostly in South and Central America, where the parasite and its vector are endemic [1]. *T cruzi* is carried in the gastrointestinal tract of triatomine bugs (Family *Reduviidae*, subfamily *Triatominae*). The parasite is transmitted to humans when the insects defecate into a bite, wound, or mucosal surface. The parasite may also be transmitted vertically from an infected mother to their child (congenital infection). When left untreated or treated too late, CD may result in cardiac or gastrointestinal disease, or both [2].

Chagas disease begins with an acute phase before moving to the chronic phase. Most acute infection patients are asymptomatic or experience nonspecific febrile symptoms [3], and therefore remain undetected. Most remain asymptomatic during the chronic phase of the disease and are called “indeterminate” cases. The chronic phase of the disease is characterized by low-level parasitemia and an IgG antibody-based immune response [3]. Between 20 to 30% of chronic CD patients become symptomatic and develop cardiac, gastrointestinal, or cardio-digestive symptoms [4].

*T. cruzi* is considered endemic in Mexico, where the WHO estimates a prevalence of nearly 0.800% [5]. However, this average misrepresents the wide prevalence variation across the country; regional CD prevalence across Mexico ranges from 0.36% to 20% [6]. CD prevalence is generally higher in rural regions [7]. Similarly, disease burden varies across Mexico, with high incidence rates reported in Yucatán (4.0 per 100,000 people), Oaxaca (2.4) and Hidalgo (2.1) [8].

### 1.1. T. cruzi Infection and Pregnancy

Congenital infection occurs in 1–5% births [9]. Adverse maternal and fetal outcomes associated with *T. cruzi* infection include low Apgar scores, poor fetal growth, low birth-weight, premature birth, and miscarriage [10]. Congenital *T. cruzi* infection is associated with myocarditis, meningoencephalitis, and/or respiratory distress [11,12,13,14]. Vertical transmission rates vary by community, though many studies report rates between 1 and 10% [11,12,13,15,16]. While overall improvement in maternal health seems to reduce complications, vertical transmission rates remain constant [10]. Congenital Chagas is estimated to account for 22% of all new *T. cruzi* infections globally [17]. If left untreated, infected infants are presumed to carry the same 20–30% lifetime risk of cardiac or gastrointestinal disease as infected adults [14]. Early detection in pregnant women is essential for identifying and treating congenital infection.

### 1.2. Diagnosis of CD

Diagnosis is made by a series of immunological or molecular tests. Acute *T. cruzi* infection may be diagnosed with direct visualization of the parasite in blood smears, among other methods. Diagnosis of chronic infection relies on the identification of antibodies against parasite antigens, as parasitemia wanes during the chronic phase. The WHO recommends that chronic CD diagnosis be made with two different serological tests, and a third if the first two are inconsistent [18]. Commonly-used serological tests include enzyme-linked immuno-sorbent assays (ELISAs), immunofluorescence assays, and immuno-blots. However, many of these tests are not readily available in most clinical settings [19]. Furthermore, many serological tests have been shown to perform differently in different geographic regions, in Mexico and across other Latin American countries [20,21,22].

### 1.3. Barriers to CD Diagnosis and Goals of Study

Diagnosis of CD is complicated by the technical shortcomings of diagnostic tests and poor access to healthcare. Furthermore, most cases are not diagnosed at birth due to the lack of accurate and timely diagnostic tests and because the disease burden is underestimated [23,24,25].

There are many obstacles to screening for *T. cruzi* infection in both endemic and non-endemic countries. A significant barrier is regional heterogeneity in disease prevalence, which creates an unclear picture of disease risk within a country or region [6]. Genetic diversity of the parasite may also create challenges for accurate diagnosis [19]. Lastly, the largest barrier is the lack of awareness of CD in both healthcare professionals and patients [26,27]. These factors all lead to less than 1% of CD patients worldwide receiving treatment [28].

Given the significant health problems associated with congenital CD and the advantages of early detection in preventing subsequent gastrointestinal and cardiac disease, it is crucial to identify cases as early as possible. The goal of this study was to screen pregnant women from endemic regions for *T. cruzi* infection seen at the Hospital General de Mexico, so that preventive treatment could be provided to women and infants.

## 2. Materials and Methods

The study protocol was approved by the National Autonomous University of Mexico (UNAM) ethics board, project number 139-2017, and by the ethics committee of the Hospital General No. HGM-DG-114-DI-2019. Written informed consent was obtained from all individuals at the time of screening.

This study took place in the Hospital General de Mexico (HGM), in Mexico City from February 2018 to February 2019. Participants were eligible for screening if they were: pregnant, 14 years of age or older, and from a Chagas-endemic Mexican state (Aguascalientes, Chiapas, State of Mexico, Guanajuato, Guerrero, Hidalgo, Jalisco, Michoacán, Morelos, Nayarit, Oaxaca, Puebla, Querétaro, Quintana Roo, Sinaloa, Tabasco, Tamaulipas, Tlaxcala, Veracruz, Yucatán, Zacatecas; based on Sistema Nacional de Vigilancia Epidemiológica/National Epidemiologic Surveillance System) or any endemic country. Additional inclusion criteria were pregnant women with heart disease or recurrent spontaneous abortions. Potential participants were excluded if they were not from an endemic region or if they declined to participate. Study recruitment occurred through direct participant enrollment at the time of prenatal care visits. In addition, flyers were posted in obstetrics ward waiting rooms notifying potential participants of the study. Recruitment and the subsequent consent process were carried out by trained study staff members.

During the study visit, a socio-demographic questionnaire designed to identify factors associated with *T. cruzi* infection was administered to each participant. Results were anonymized and recorded in an electronic REDCap database [29,30]. The information collected included clinical and epidemiological characteristics such as age, sex, state of origin, household characteristics, exposure to the triatomine bug, and questions about previous blood transfusions or organ transplantation.

Venipuncture was performed during the initial study visit, and sera were stored at −20 °C. An initial screening was done with the ELISA test using antigens grown in-house [31]. All samples were additionally sent to the blood bank of the General Hospital for confirmatory testing with either the *ABBOTT* PRISM Chagas chemiluminescent immunoassay or the Bio-Rad Chagascreen ELISA. The ABBOTT and Bio-Rad tests are commonly used serological tests for *T. cruzi* infection. Considering the variability by location of tests’ performance [20,21,22], this in-house ELISA was chosen as a screening method because its antigens were grown using local parasite strains, as detailed below.

All positive or indeterminate patients by any of the mentioned tests were then tested with InBios Chagas Detect Plus rapid test, Accutrack Chagas Recombinant micro-ELISA test and qPCR (Figure 2). Forty samples that had negative results with the “in house” ELISA and the tests run by the hospital blood bank (i.e., the *ABBOTT* PRISM *Chagas* assay or the Bio-Rad Chagascreen ELISA), were randomly selected to be tested with the Inbios Chagas Detect Plus rapid test.

### 2.1. “In-House” ELISA Test Antigen Preparation

*Trypanosoma cruzi* Queretaro strain TBAR/MX/0000/Queretaro (“Qro”) epimastigotes were maintained in liver infusion tryptose culture medium (LIT), supplemented with 10% heat-inactivated fetal bovine serum in axenic conditions at 28 °C [32]. For antigen preparation, epimastigotes were washed in cold PBS, centrifuged, and resuspended in 50 mM Tris-HCl at pH 7.4 with protease inhibitors (aprotinin: 10 µg/mL, leupeptin: 2 µg/mL and benzamidine: 1 mM). They were sonicated in cold with 6 cycles of 1 min. Effective cell lysis was verified through light microscopy. The samples were centrifuged at 30,600× *g* at 4 °C for 1 h. Supernatants were collected and their protein quantified by Bradford assay. Aliquots of 20 µL were stored at −70 °C until testing.

### 2.2. “In House” ELISA Test Antigen Detection

The in-house ELISA test was made with 0.6 μg of prepared *T. cruzi* Qro-strain antigen diluted in 100 μL of PBS per well and fixed one hour on a 96 well EIA/RIA plate at room temperature (modified by Ryan et al., 2002) [33]. The antigen was then discarded and blocked with 200 μL of blocking solution for one hour at room temperature. Patient sera were analyzed in triplicate. They were diluted 1:100 in blocking solution and 100 µL were added per well and incubated for two hours at room temperature. The sera were discarded, the ELISA plate was washed 4 times with 300 µL PBS-Tween per well in an automated plate washer. The peroxided secondary antibody (HRP-rabbit anti human IgA, IgG, IgM, Kappa, Lambda) was diluted 1:12,000 and 100 µL were added per well for 30 min at room temperature. The secondary antibody was discarded by washing and tetramethylbenzidine (TMB) peroxidase substrate and peroxidase solution B were mixed in equal volumes and added using 100µL per well. The plate was incubated with this solution for 30 min at room temperature, protected from light. The enzymatic reaction was stopped using 100 µL of phosphoric acid 1 M. Results were obtained by examining the plate in an EIA plate reader at 450 nm. The Cut-Off value was determined by the following formula: 3X standard deviation sample + negative control average. The results were calculated by subtracting the Cut-Off value from the average of the three serum samples. The samples were considered positive if their OD values were at least 10% higher than their Cut-Off. The samples were negative if their OD values were at least 10% lower than their Cut-Off and indeterminate if OD values were within 10% of the Cut-Off value.

### 2.3. Accutrack Chagas Recombinant MicroELISA Test, Lemos Laboratory, Buenos Aires, Argentina

This is an immunoenzymatic assay with recombinant antigens and was used according to the technical specifications in the product [34].

### 2.4. Chagas Detect^TM^ plus Rapid Test

This is a rapid immunochromatographic strip assay for the qualitative detection of human IgG antibodies to *T. cruzi* in human serum. This rapid test was used according to the technical specifications in the product [35].

### 2.5. qPCR and DNA Blood Extraction

PCR was conducted on samples with positive results, indeterminate results, and four random negative samples. Blood samples (10 mL) were mixed with one volume of Guanidine Hydrochloride 6 M, EDTA 0.2 M buffer, pH 8.00 (GE) and boiled for 10 min at 98 °C. Blood treated with GE (GEB) and serum samples were processed for DNA extraction using the High Pure PCR Template Preparation kit (Roche Diagnostics Corp., Indiana, IN, USA), following the recommendations of the manufacturer. Binding buffer (200 μL), proteinase K (20 μL) and 300 μL of the sample (GEB or serum) were mixed and incubated during 10 min at 70 °C. Isopropanol (100 μL) was added, mixed and the sample was placed in a filter tube assembled in a collection tube and centrifuged at 8000× *g* for 1 min. The filter tube with the sample was washed once with inhibitor removal buffer (500 μL) and twice with washing buffer (500 μL) and centrifuged. The filter tube was dried by centrifugation at 8000× *g* for 1 min. Samples were eluted with 100 μL of elution buffer at 70 °C [34]. Samples were extracted in duplicate.

### 2.6. Multiplex Real-Time PCR Assay

Real-Time multiplex PCR for identification of *T. cruzi* in the biological samples was performed using TaqMan probes reported to detect satellite DNA (SatDNA) of the parasite, and human RNAse P gene was used for quality control (Table S1). All PCR reactions were carried out with 5 μL of DNA in a final volume of 20 μL. All PCR reactions were carried out with 5 μL of DNA in a final volume of 20 μL. The reaction mixture included: 10 μL of Universal PCR Master mix 2× (Applied Biosystems), 1 μL of cruzi1 and cruzi2 oligonucleotides (10 μM), 0.2 μL of cruzi3 (5 μM), 0.5 μL of RNAse P 20× (Applied Biosystems) and 1.3 μL of RNAse-free water. Optimal cycling conditions for the qPCR assay were initially 10 min at 95 °C, followed by 40 cycles at 95 °C for 15 sec and 58 °C for 1 min (with data collection) in an Applied Biosystems (ABI 7500 Fast) device [36]. Samples were analyzed in duplicate.

### 2.7. Statistical Analysis

Descriptive statistics were conducted using the statistical software Stata 14.38 [37].

## 3. Results

**Figure 1:**
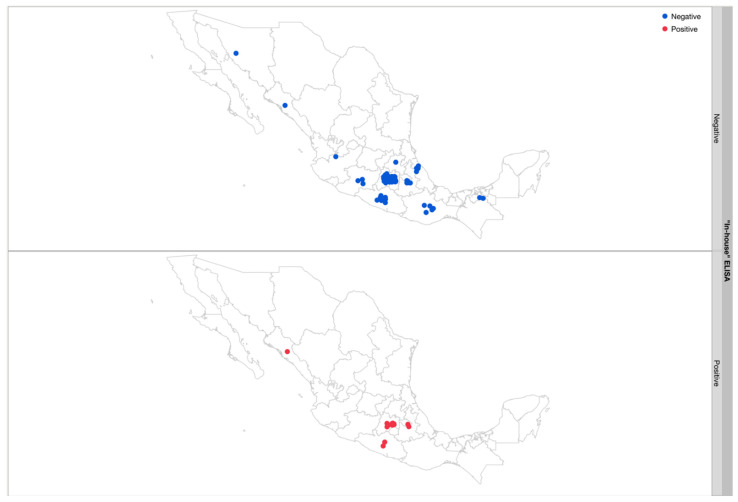
States of origin of patients with positive and negative results from the CD screening test (“in-house” ELISA) used at HGM.

Overall, 150 patients consented to participate and provided samples for analysis, mean age 26.8 (SD 6.4). Most (97.9%) came from Mexico, primarily Mexico City (40%), State of Mexico (31.3%), Guerrero (6%), and Puebla (4.6%); the state of origin for Mexican patients along with their screening test result may be found in Figure 1. Two patients were from Venezuela (1.3%) and one from Colombia (0.6%). All 147 of the Mexican participants completed the survey. Few had heard of CD; 25 (16.9%) and 23 (15.54%) had seen a triatomine, and only one (0.68%) reported knowledge of being bitten by a triatomine.

Of the 150 participants tested, 17 (11.3%) were positive and 5 (3.3%) were indeterminate by the in-house ELISA screening test. Of these 22, 14 (63.6%) were positive by the InBios rapid test, 2 (9.1%) by Accutrack, 1 (4.5%) by the blood bank tests and 9 (40.1%) were positive by PCR.

**Figure 2:**
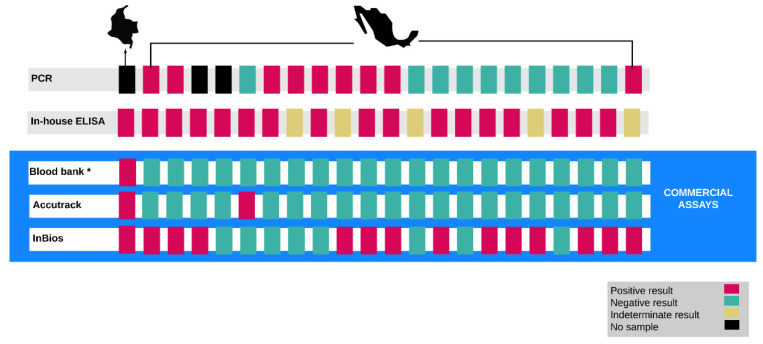
CD test results of participants with positive or indeterminate results by in house ELISA. * Blood bank tests include: ABBOTT PRISM Chagas assay or BioRad Chagascreen ELISA. Of these, 12 were positive by the WHO-defined criteria of having at least two different serological tests in agreement; nine were positive by qPCR. Three participants could not successfully be reached for further blood draws, and so no sample was available to run PCR on these patients. The country of origin of these patients is indicated at the top of the figure (Colombia, left; Mexico, right).

Regardless of screening test results, two (1.3%) were positive by the Accutrack ELISA, 23 (15.3%) were positive by InBios, 9 (6%) were positive by PCR, and 1 (0.7%) by Chagascreen. Sixteen samples (10.7%) were positive by at least two tests (Figure 2). Five (3.3%) were positive by the in-house ELISA, the InBios rapid test, and at least one other test. Thirty (20.0%) were positive by at least one test (Table 1).

Among the positive tests, results were highly discrepant across the different tests. Nine patients (6%) were positive by the most specific test alone (PCR) (Figure 2). When compared to serologic tests, none of the PCR-positives were positive by Accutrack ELISA. Even though 23 (15.3%) of the patients tested by the FDA-approved InBios rapid test were positive, only six of these cases were also positive by PCR.

**Figure 3:**
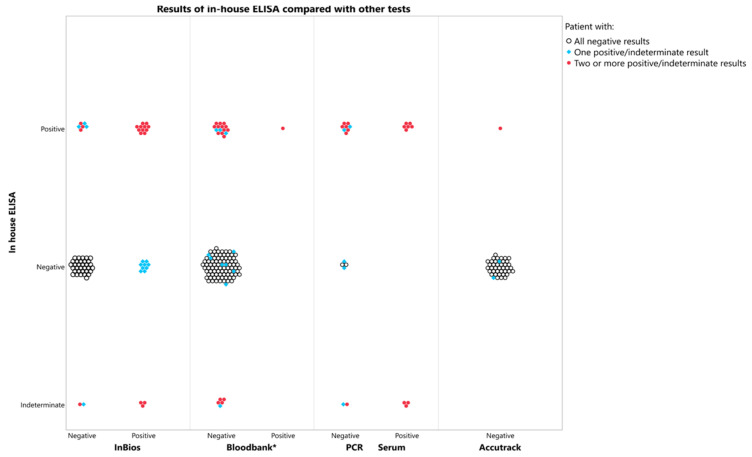
Test concordance with in-house ELISA results. * Blood bank tests include: ABBOTT PRISM Chagas assay or BioRad Chagascreen ELISA.

Using the WHO diagnostic criteria for chronic CD of agreement between at least two different *serological* assays (here, the in-house ELISA, the Accutrak ELISA, the InBios rapid test, the Abbott Prism, or the BioRad Chagascreen), 12 samples (8%) were positive; of these 12 samples, 4 were positive by PCR (Figure 3).

Nine of the 23 samples positive by the Inbios rapid test, tested negative using all other diagnostic tests.

## 4. Discussion

Our site is a main reference hospital in Mexico City and serves local residents as well as women referred for high-risk pregnancies. Despite the low reported awareness of CD and its insect vector among our sample population, our results indicate ongoing undetected CD transmission in Mexico. The in-house ELISA, developed in Mexico with TcI strain Qro, found 11.33% of the sample population to be positive. Five positive and one indeterminate result on the screening test (in-house ELISA) could have been overlooked by commercial tests, as what happened with the Accutrack and blood bank ELISAs. Further testing with additional commercial assays and larger sample sizes could clarify this. The Inbios rapid test, used on a subset of 64 samples, found 35.94% (n = 23) of this subset to be positive. A total of 39.13% of the 23 samples tested with PCR were found to be positive. Though these results may vary, they all attest to continuing maternal infection with *T. cruzi.*

There has been no country-wide seroprevalence study in Mexico that focuses on pregnant women, though a 2019 systematic review and meta-analysis of prevalence studies from various regions of Mexico, 2006–2017, found an estimated seroprevalence of 2.21% among pregnant women [38]. In Mexico City specifically, a seroprevalence among pregnant women of 4.12% was found in 2012 (n = 1448) [39]. In Palenque and Poza Rica, two other urban cities in Mexico, rates of 5% and 3.5% were found among pregnant women. Some studies in other regions report rates as high as 17% [40]. Our study reinforces the importance of screening pregnant women for *T. cruzi* infection, as they have a higher seroprevalence than the general population: A 2005 study using blood bank specimens found a 0.37% seroprevalence in Mexico City [41]; compare this to the 2.12% seroprevalence found in pregnant women [38].

Currently, pregnant patients are not routinely screened for *T. cruzi* infection, despite the benefits for their own well-being as well as their child’s. Pregnant patients identified as positive can then have their babies tested early for CD. Early detection is particularly important among infants, as it poses a unique opportunity to provide treatment that definitively clears the infection. Two pharmaceutical treatments for CD exist, Benznidazole and Nifurtimox, though both cause severe side effects in adults and do not ameliorate cardiac symptoms in chronic patients [42,43]. Both drugs have a less severe side effect profile in young children, making timely diagnosis of congenital CD crucial [44]. It is particularly important in non-endemic settings where re-infection is not expected. At-risk populations should undergo greater monitoring and surveillance for CD.

Strengthening access to healthcare is crucial to obtaining adequate screening for CD. One suggested approach at the country level is developing initiatives tailored to the local health system and in alignment with general health goals and programs. Such is the case of Mexico and the Specific Action Program for the Prevention and Control of Chagas Disease 2013–2018, which actively engaged in activities to eliminate transfusion and congenital transmission [8]. Our study showed that while overall disease prevalence is low, in selected groups, there is a need to initiate screening and follow-up processes when appropriate. Another primary care-based screening pilot program in Boston found 3.8% positive screening tests, demonstrating the feasibility of incorporation of CD screening in primary care in non-endemic settings [45]. Custom-made interdisciplinary initiatives that align with local goals and health projects are feasible, effective, and should be promoted to screen as many people as cost-effectively as possible in primary care settings.

Effective *T. cruzi* screening does however rely on reliable testing methods. Our study made use of many different assays, though each did not identify the same samples as positive. The varying abilities of serology tests to detect infections among the same set of patients could be due to the different *T. cruzi* strains used to develop and produce the tests and by the wide antibody response [46]. The *T. cruzi* species has six human-infective phylogenetic categories, called Discrete Typing Units (DTUs): TcI through TcVI. DTUs have distinct but overlapping geographic ranges and differing clinical outcomes and ecological characteristics [47]. TcI and TcIV have been detected in Mexico, with TcI being the most prevalent [46,48,49]. A serology test may best detect infections of the same lineage from which its antigens were cultivated. CD in the US and Mexico is mostly caused by TcI lineage parasites [50]; however, not all tests in our study were developed using this lineage. We did choose our in-house ELISA as a screening test because it was developed using a TcI lineage strain. Our study showed notable variation between all of the serological testing methods used, and while certainly not conclusive, it supports the idea that serological tests have varying efficacies among different populations due to the strain to which they were exposed. Our study also highlights the potential need for standardized guidelines for diagnosis and testing according to the predominant lineage. Previous work from our team has highlighted these needs as well [6].

Promising tests that could be incorporated into future studies include molecular assays for detection of congenital infection in babies. There is also a need to develop highly specific serological tests that can be easily implemented in community settings. This opens an important window of opportunity for the detection of CD in women and an early treatment for children, at a stage when the disease is curable with a low side effect profile.

### Study Limitations

This study has limitations. Sample size restricts in-depth analysis of test performance and patient characteristics. While the diagnostic algorithm was completed according to current clinical practices at the institution, additional tests such as PCR, Inbios rapid test, and Accutrak Recombinant ELISA were not performed on all participants that had resulted negative according to the diagnostic algorithm due to resource constraints. Three patients with positive “in-house” ELISAs were not available to provide adequate blood samples for PCR testing. Moreover, genotyping was not conducted and therefore diagnostic performance cannot be associated with genetic characteristics of the parasite.

Despite our study limitations, we provide evidence for the necessity of prenatal screening of women from Chagas-endemic areas, and of the importance of revising current diagnostic approaches for *T. cruzi* infection.

## 5. Conclusions

We provide evidence of ongoing *T. cruzi* infection in pregnant women in Mexico City, at a rate higher than that found by other studies in other Mexican states. Screening in the prenatal setting is of particular importance given the opportunity to treat both mother and infant. However, we encountered high variability between the serological screening tests used, which possibly highlights the need for region-specific *T. cruzi* assays. The lack of a reliable test for CD remains a diagnostic challenge in both endemic and non-endemic countries.

## Figures and Tables

**Table 1:** Positive results by test type.

	Test	Indeterminates ^1^	Positives ^2^	% Positive ^3^
Blood bank tests	ABBOTT PRISM Chagas assay	0/42 = 0%	0/42 = 0%	1/150 = 0.67%
	BioRad Chaga-screen ELISA	0/108 = 0%	1/108 = 0.93%	
Laboratory tests	In-house ELISA	5/150 = 0.3%	17/150 = 11.3%	17/150 = 11.3%
	Inbios Chagas Detect Plus	N/A	23/64 = 36.0%	23/150 = 15.3%
	Lemos Labs. Accutrak Recombinant ELISA	N/A	2/25 = 8%	2/150 = 1.3%
	qPCR	N/A	9/23 = 39.1%	9/150 = 6%

^1^ Samples yielding indeterminate results with this test, out of all samples tested with this test. ^2^ Samples yielding positive results with this test, out of all samples tested with this test. ^3^ Positive samples by this test as a percentage of all samples.

## Data Availability

All datasets available upon request to corresponding author.

## Supplementary Materials

The following are available online at https://www.mdpi.com/article/10.3390/tropicalmed7010008/s1, Table S1: Probes used in Multiplex qPCR Assay.
