# Supplementary material for: Chagas Disease in Pregnant Women from Endemic Regions Attending the Hospital General de Mexico, Mexico City
Source: Trop Med Infect Dis. 2022 Jan 11;7(1):8. doi: 10.3390/tropicalmed7010008 (PMC8779423; doi:10.3390/tropicalmed7010008)
Supplement: Supplementary file 1 [file tropicalmed-07-00008-s001.zip › tropicalmed-1491786-supplementary.pdf]

Tropical Medicine and Infectious Disease

Supplementary material of Chagas Disease in Pregnant Women from Endemic Regions Attending the Hospital General de Mexico, Mexico City

Table S1. Probes Used in Multiplex qPCR Assay.

Target Sequence	Oligonucleotide	Sequence (5′–3′)
T. cruzi SatDNA	Cruzi1	ASTCGGCTGATCGTTTTCGA
	Cruzi2	AATTCCTCCAAGCAGCGGATA
	Cruzi3	FAM-CACACACTGGACACCAA-NFQ-MGB
RNAse P	RNAse P Assay	unknown
